# PSD microscopy: a new technique for adaptive local scanning of microscale objects

**DOI:** 10.1186/s40638-017-0063-5

**Published:** 2017-10-24

**Authors:** Mehdi Rahimi, Yantao Shen

**Affiliations:** Department of Electrical and Biomedical Engineering, University of Nevada, Reno, 1664 N. Virginia Street, 89557 Reno, NV USA

**Keywords:** Position-sensitive detector, Microscopy, Adaptive method, Complicated objects, Scanning patterns, Local scanning, SPM, Indiscrete objects, Micro-objects

## Abstract

A position-sensitive detector/device (PSD) is a sensor that is capable of tracking the location of a laser beam on its surface. PSDs are used in many scientific instruments and technical applications including but not limited to atomic force microscopy, human eye movement monitoring, mirrors or machine tool alignment, vibration analysis, beam position control and so on. This work intends to propose a new application using the PSD. That is a new microscopy system called scanning PSD microscopy. The working mechanism is about putting an object on the surface of the PSD and fast scanning its area with a laser beam. To achieve a high degree of accuracy and precision, a reliable framework was designed using the PSD. In this work, we first tried to improve the PSD reading and its measurement performance. This was done by minimizing the effects of noise, distortion and other disturbing parameters. After achieving a high degree of confidence, the microscopy system can be implemented based on the improved PSD measurement performance. Later to improve the scanning efficiency, we developed an adaptive local scanning system to scan the whole area of the PSD in a short matter of time. It was validated that our comprehensive and adaptive local scanning method can shorten the scanning time in order of hundreds of times in comparison with the traditional raster scanning without losing any important information about the scanned 2D objects. Methods are also introduced to scan very complicated objects with bifurcations and crossings. By incorporating all these methods, the new microscopy system is capable of scanning very complicated objects in the matter of a few seconds with a resolution that is in order of a few micrometers.

## Background and introduction

A position-sensitive device (PSD) is a sensor that is capable of tracking laser beam on its surface. The four current outputs of the sensor would give the position of the light incident. PSDs have received attention for a long time [1]. They are used in many applications including but not limited to atomic force microscopy (AFM) [2, 3], spectroscopy [4, 5], particle tracking [6, 7], image position sensing [8, 9], displacement sensing [10], robot calibration [11] and many other applications. Also, different methods of improving the accuracy of the PSDs have also been discussed by researchers [12–14].

In this work, we have used some of the PSD characteristics to develop a new kind of microscopy system. Using this system, a user can put a small object (something that is even as small as a few micrometers) on the surface of the PSD, and then by using the system, the dimensions of the objects with a very good accuracy and precision can be determined. It is important to note that there is no limitations to the object shape or property used in this research. Any solid object, even transparent objects such as glass, can be used. Non-solid objects can be scanned using this method too, provided that they can be placed on a glass that covers the whole PSD sensor.

To achieve this, we first had to improve the accuracy of the PSD. “Measurement accuracy improvements” section talks about how we did this improvement. A PSD sensor can be affected by numerous factors including noise, distortion, rotation and vibration. The first part of this work addresses these challenges. All of the noises that are affecting the system are analyzed one by one, and preventive measures have been taken to lessen their effect as much as possible. The distortion problem of the PSD is also discussed in this section. A method is used to rectify the distortion effect of the PSD. Various validations have been done to make sure that the effect of noise and distortion is mitigated. Other challenges such as *X*–*Y* mismatch and rotation correction are also discussed in this section. At the end and based on the new improved PSD system, a microscopy system is proposed. The system works in this manner that a user can put an object on the surface of the PSD, and by scanning the area of the PSD, one can find the object and its dimensions.

In “Adaptive local scanning” section, we continue to improve the microscopy system by developing and implementing an adaptive local scanning for the PSD. Although a traditional scanning (or raster scanning) needs to scan every pixel of an area to find the object on the surface of the PSD, we suggested an adaptive local scanning to minimizing the time needed for this scanning. This is done by first using an initial scanning which would roughly find the position of the object and then a fine scanning that uses a sinusoidal pattern to scan the boundaries of the object. The pattern would follow the curvature of the object and would even recognize bifurcations and crossings. This smart algorithm would change the amplitude and frequency of the sine wave automatically to match the circumstances of the object. A very sophisticated algorithm would use an Archimedean spiral to find missed points of the curve and to recognize all the crossings and bifurcations. Eventually, the algorithm would scan the whole object no matter how many branches or crossings it has. The algorithm even has a subroutine for thick objects that would fit in the sinusoidal pattern and therefore can scan these objects much faster than by going around them. At the end, a Fourier curve fitting is employed to get even better results from the reading we got from the scanning pattern. Extensive implementation results demonstrated that the fitting can reduce the scanning error to an almost half of the original value. It also validates that our comprehensive and adaptive local scanning method can shorten the scanning time in order of hundreds of times in comparison with the traditional raster scanning without losing any important information about the scanned object.

By putting all these methods together, we can have a microscopy system that can scan and find the dimensions of a very small object in the matter of a few seconds with a very high accuracy and precision.

## Measurement accuracy improvements

To improve the measurement accuracy of a lateral effect PSD system, in this part, different noises are identified, analyzed and eliminated by the signal averaging. In addition, effects of *X*–*Y* mismatch and rotations are corrected. Finally, a distortion rectifying algorithm effectively solving pincushion-type radial distortions is developed. Both simulation and experimental results verify the effectiveness of the developed accuracy improvement methods. Such a PSD system with high reliability and accuracy can be further used for a proposed scanning PSD microscopy. The microscopy is based on the vanishing effect and the multi-channel photocurrent feedback mechanism of the PSD. The vanishing effect is related to the four zero or low photocurrent outputs when the laser beam is blocked by meeting the object on the PSD surface. Several methods for detecting the exact positions where the blockings of the laser occur are proposed and implemented. Once the blocking positions of a scanning laser beam pattern are determined, the 2D of the micro-object on the PSD surface can be accurately measured. Preliminary results on accurately and rapidly measuring both opaque and transparent objects demonstrate the performance of the proposed microscopy system.

### The lateral effect PSD

**Fig. 1:**
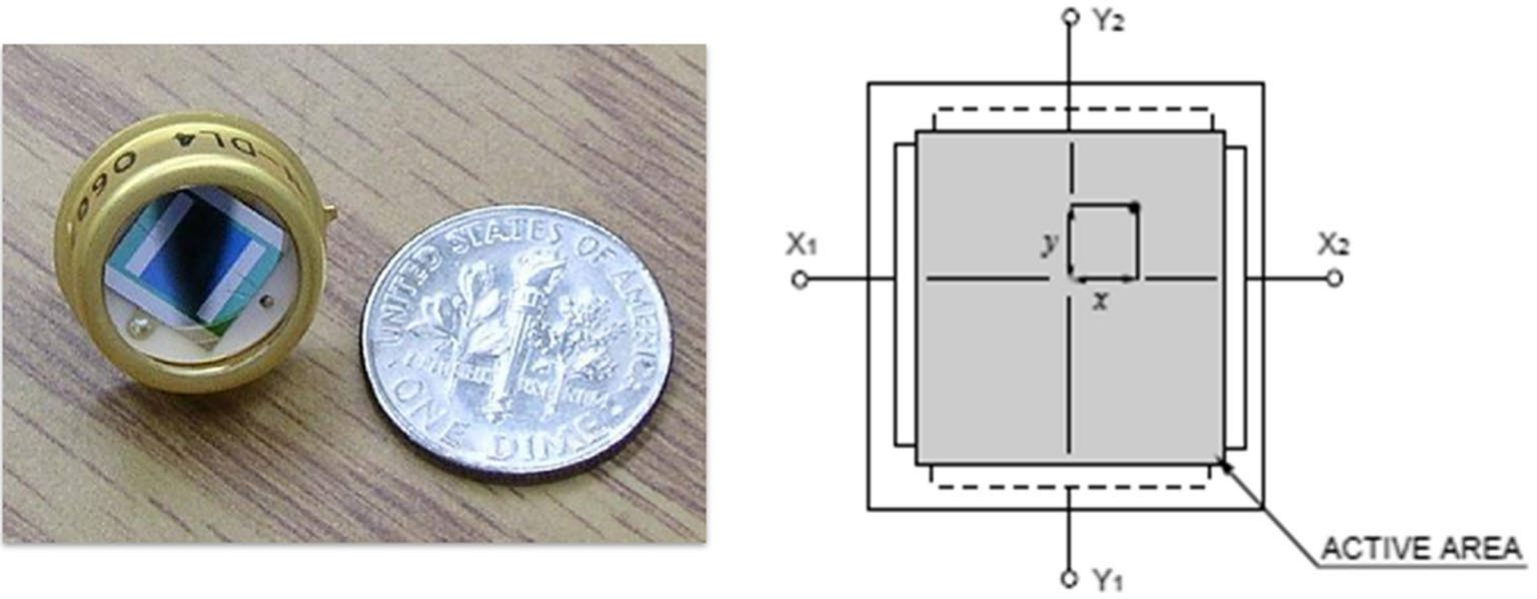
Photograph of lateral effect PSD chip and a schematic illustration showing the two electrodes that are mounted on opposite sides of the PSD to determine *X*-axis and *Y*-axis direction are shown here

To implement our improvement methods, we have chosen a duo-lateral effect PSD (OSI Optoelectronics DL-4S) for building up the test bed and modeling. The lateral effect PSD is shown in Fig. 1 is a 2D sensor with 4 mm × 4 mm active area. In a lateral effect PSD, the relative two-dimensional position of light beams such as a laser beam on the active surface of the chip can be expressed as:

1 $$\begin{aligned} X= \,& {\frac{I_{x1} - I_{x2}}{I_{x1} + {I_x2}}} \nonumber \\ Y= \,& {\frac{I_{y1} - I_{y2}}{I_{y1} + I_{y2}}} \end{aligned}$$

where *X* represents the relative position of the laser spot on the *X*-axis and *Y* is the relative position on the *Y*-axis. Also $I_{x1}$, $I_{x2}$, $I_{y1}$, $I_{y2}$ are photocurrents measured in the direction indicated by the index, which in the following experiments are called Channel *A*, *B*, *C* and *D*, respectively.

A very important property that has been used several times in the following experiments is that because of the structure of the PSD, opposite channels have inverse correlation; meaning that when one of them increases, the other one decreases. This effect is easily shown in Fig. 1.

The major advantage of the lateral type PSD is that the accuracy of the output is not affected by the laser spot profile or its intensity distribution. The positional resolution of about 0.5 μm is sufficient for positioning in micro-level. Another outstanding property of this type is the position linearity over relatively large areas of the active surface of the chip. This is important for our task since it allows us to keep errors at a low level during filtering, rectifying, and mapping processes. The signal conditioning circuit of the PSD can be employed and found in [15].

### Accuracy improvement methods and experimental validation

#### Noises and filtering

**Fig. 2:**
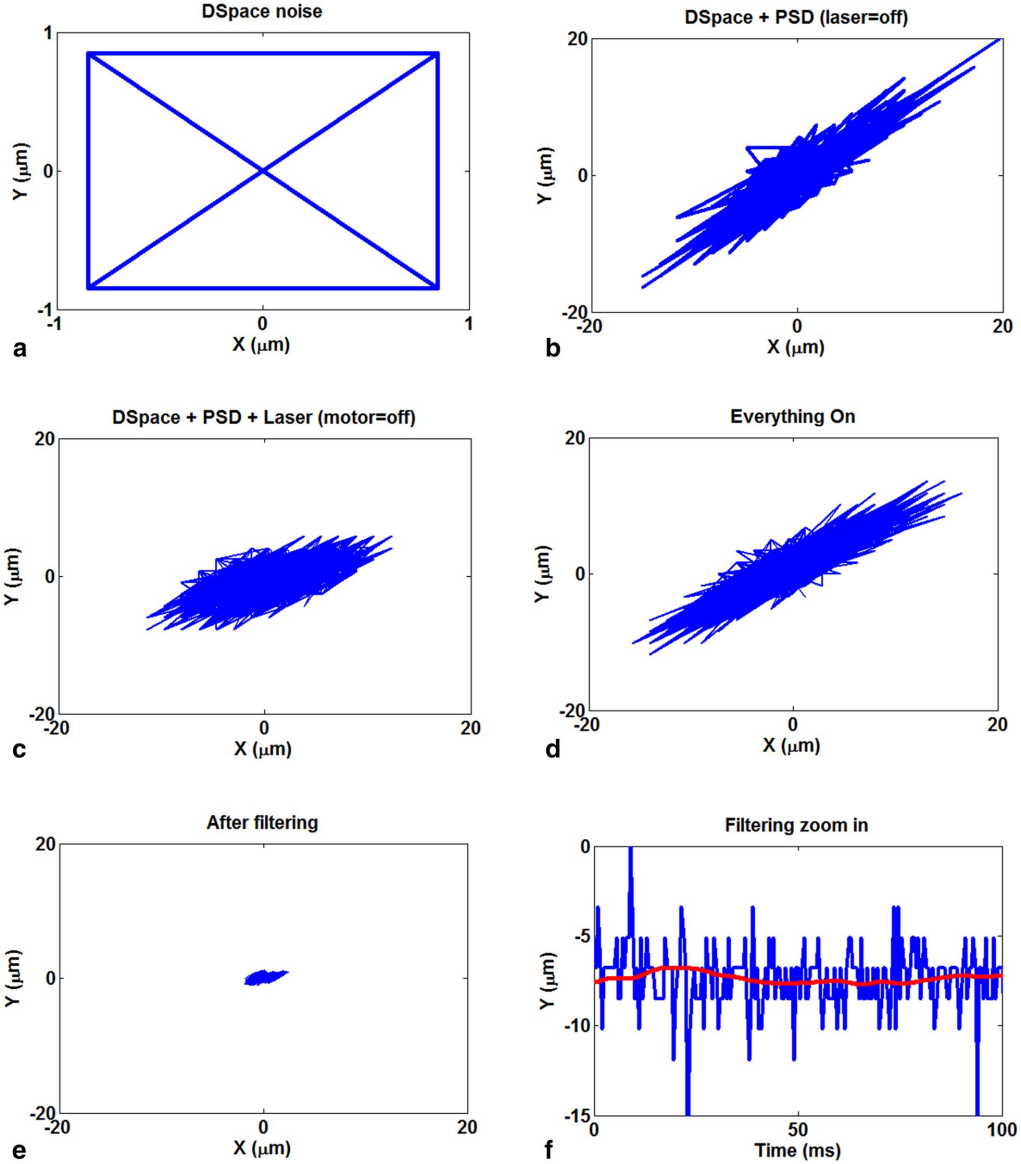
**a** Noise that dSPACE produces results in < 1 μm movement in each direction; **b** noise of the system when there is not a laser beam on the PSD; **c** noise of the whole system except the noise of the micro-manipulator motor; **d** noise that the whole system produces when everything is on can be seen as an almost 15 μm vibration in each direction; **e** filtering has reduces the noise movement from 15 to 1–2 μm vibration in each direction; **f** a comparison of actual signal (blue) with the filtered signal (red graph in the middle) in an experiment

There are different sources of noise for the whole system. The micro-manipulator motor controls the laser, the ambient light on the PSD, the PSD board and its connections, the amplifiers, the probe, the dSPACE panel and control board—which is used to get the data from the PSD board—and finally any movements in the area that may cause the PSD or the laser to shake. Luckily, this part was set up on an anti-vibration table that was able to absorb movement noises to some acceptable degree, but there were still lots of other noises. A set of tests were designed and run to measure each of these noises and their impact on the system. The dSPACE noise is the most negligible noise as it produces a noise that results in < 1 μm movement in each direction which is shown in Fig. 2a. This noise is probably because of the dSPACE internal oscillator crystal. Another test measures the noise that the dSPACE and the PSD produce together but without any laser beam, meaning that it just shows the effect of ambient light and the noise of the PSD board. This is shown in Fig. 2b. We also noticed that the micro-manipulator motor that moves the laser has a considerable noise. This can be seen when comparing Fig. 2c, d. And finally, the noise that the whole system produces when everything is on and working is shown in Fig. 2d. Notice that in all of these experiments, the motor is not receiving any command to move so the noise of the system is causing a vibration as high as almost 1 μm in each direction. Note that all the figures in Fig. 2 have been centered for easier comparison.

**Fig. 3:**
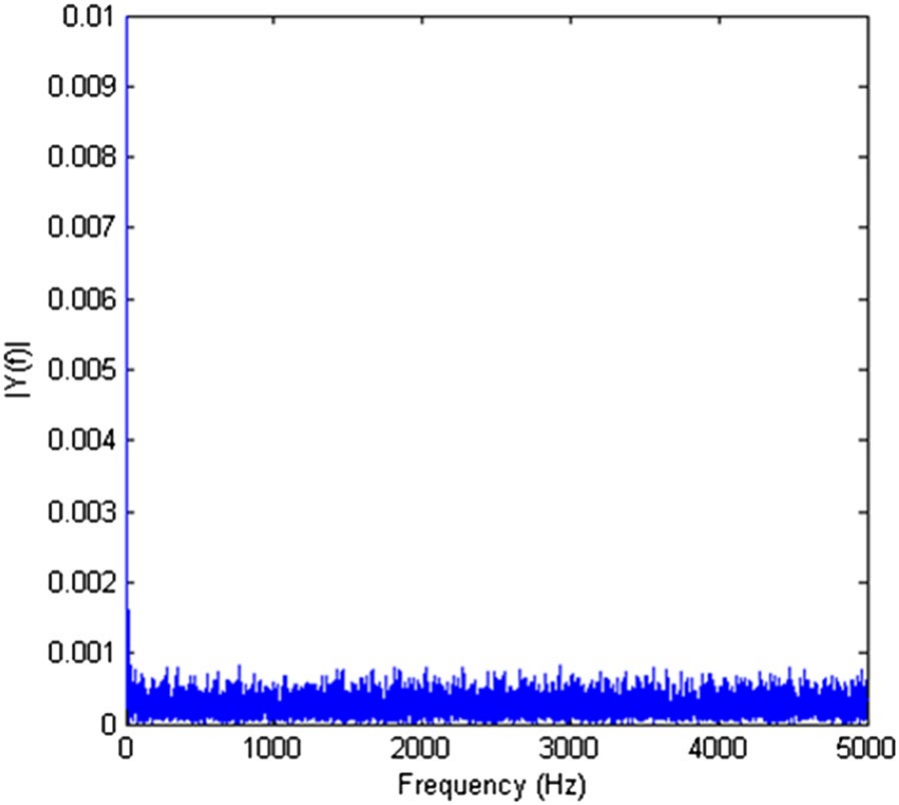
FFT of the noise of the whole system does not show any particular frequency for the noise to filtering it out

Fast Fourier transform (FFT) can be used as a powerful tool for analyzing and measuring noise signals as we can effectively measure the frequency content. Unfortunately, the only problem for this system is that, as it is shown in Fig. 3, the noise does not occur in any special frequency. In other words, the system has a noise that is more similar to white noise. This means that the low-pass filters, band-pass filters or similar approaches are not applicable for filtering out the noise of this system. But as in this experiment, the DC part of the signal is more important to us, and we can use other methods to eliminate the noise. One can just use an AC to DC converter, but that would remove useful information too.

Signal averaging is a signal processing technique applied in the time domain, intended to increase the strength of a signal relative to noise that is obscuring it. By averaging a set of replicate measurements, the signal-to-noise ratio, *S*/*N*, will be increased [16]. To prove this, suppose the noisy signal *v*(*k*) is sampled every *T* seconds:

2 $$\begin{aligned} v(kT)=v_s(kT)+V_{\text{noise}}(kT) \end{aligned}$$

If *N* partitions are composed, the averaged signal becomes:

3 $$\begin{aligned} y(kT)= & \sum _{i=1}^{N} v_s^i(kT) + \sum _{i=1}^{N} v_{\text{noise}}^i(kT) \nonumber \\&\forall k=1,2,\ldots ,M \end{aligned}$$

If the partitions are perfectly aligned and the signal is truly periodic, the desired signal adds up:

4 $$\begin{aligned} \sum _{i=1}^{N} v_s^i(kT) = N v_s(kT) \end{aligned}$$

However, for Gaussian noise with zero mean and a standard deviation $\sigma _n$ (which also equals its rms value), we obtain

5 $$\begin{aligned} \sum _{i=1}^{N} v_{\text{noise}}^i(kT) = {\sqrt{N \sigma _n^2}} = {\sqrt{N \sigma _n}} \end{aligned}$$

Taking the ratio of (4) and (4), we can find the signal-to-noise ratio (SNR) after averaging *N* partitions:

6 $$\begin{aligned} {\text{SNR}}_N={\frac{Nv_s(kT)}{{\sqrt{N}} \sigma _n}} = {\sqrt{N}} \times {\text{SNR}}_1 \end{aligned}$$

Thus, we get an ${\sqrt{N}}$ improvement in the SNR.

**Fig. 4:**
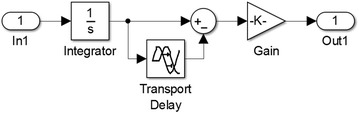
Filter model in the Simulink

As the previous figures have shown, the mean of the noise is zero and we know that signal and noise are uncorrelated, so signal averaging is the perfect solution in this case. The only disadvantage of this method is that it produces some amount of delay based on the period we use for averaging. The Simulink^®^ modeling of this filter is shown in Fig. 4.

Here gain block gives us 1/*T* where *T* is the time delay of transport delay block in seconds. The result shows significant improvement in noise reduction. The signal before filtering shows a vibration of about 15 μm in each direction, but after the filtering, the movement is limited to 1–2 μm in each direction. This has been achieved without losing actual data. The filtered signal follows the actual signal perfectly and the maximum delay is < 50 ms.

#### *X*–*Y* mismatch and rotation correction

**Fig. 5:**
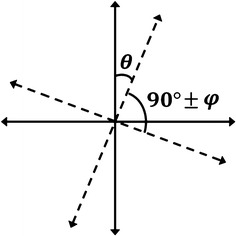
The effect of rotation and skew matrices that were discussed in “*X*–*Y* mismatch and rotation correction” section is shown here. The relationship between $\theta$ and $\varphi$ can be seen. It is important to note that these two factors work simultaneously and can have a combined effect

One of the problems with calibrating and actually using the data we get from the PSD is that the *X*- and *Y*-axes are not made perfectly perpendicular with each other in the PSD and also the PSD is not parallel with the motor that moves the laser. The first problem results in data that are not matched in just one direction and the second problem results in data that seem to be rotated a little bit. Notice that if the micro-manipulator axes were not perpendicular too, it would result in the same problem as the first one. To match the two Cartesian coordinates that are on the PSD chip surface and on the position sensing software interface, a rotation matrix $$\left({\begin{matrix} \cos \theta &{}-\sin \theta \\ \sin \theta &{}\cos \theta \end{matrix}} \right)$$ has been used to correct the mismatch between the two coordinates, where $\theta = 2.29$ is the calibrated mismatch angle between the coordinates. Similarly, to solve the problem of the *X*- and *Y*-axes not being perfectly perpendicular on the surface of the PSD, a skew matrix $$\left({\begin{matrix} 1&\tan \varphi \\ 0&1 \end{matrix}} \right)$$ was used, where $\varphi = -\,2.864$ was calculated. These corrections are shown in Fig. 5.

#### Distortion rectifying

The *X*- and *Y*-axes on the PSD have radial distortion. This means there is a deviation from the rectilinear projection. The type of radial distortion that the PSD has is called pincushion distortion. In pincushion distortion, the visible effect is that scanning lines that do not go through the center of the image are bowed inwards, toward the center of the image. To correct this distortion, different approaches were researched and employed. As an example, research in [17] developed a correction method for this distortion, while in this work the Brown’s distortion model [18, 19] was modified and employed.

7 $$\begin{aligned} x_{\text{u}}= \,& (x_{\text{d}} - x_{\text{c}})\left( 1+K_1 r^2 + K_2 r^4 + \cdots \right) \nonumber \\ y_{\text{u}}= \,& (y_{\text{d}} - y_{\text{c}})\left( 1+K_1 r^2 + K_2 r^4 + \cdots \right) \nonumber \\ r= \,& {\sqrt{(x_{\text{d}} - x_{\text{c}})^2 + (y_{\text{d}} - y_{\text{c}})^2}} \end{aligned}$$

where $(x_{\text{u}}, y_{\text{u}})$ is the undistorted point, $(x_{\text{d}}, y_{\text{d}})$ is the distorted point, $(x_{\text{c}}, y_{\text{c}})$ is the center of distortion and $K_n=n$th is radial distortion coefficient. For this distortion, $K_1 = -\,0.62$ and $K_2 = 0.28$ were measured.

#### Mapping validation

**Fig. 6:**
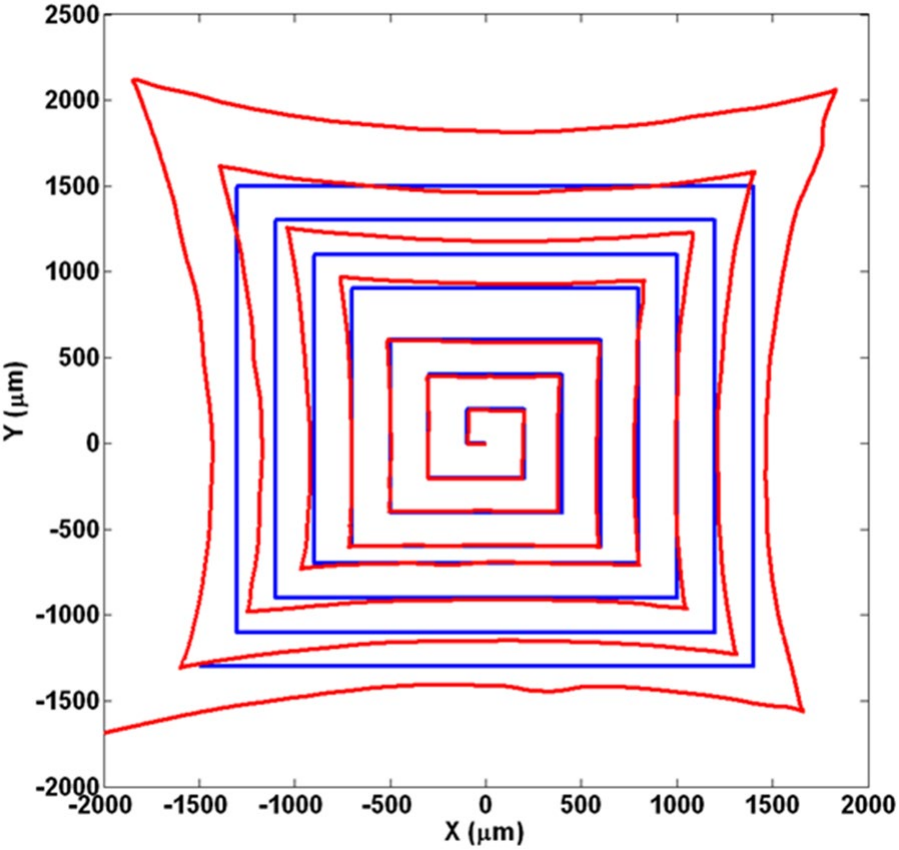
A meander pattern was used to determine the distortion (the red bigger graph). The blue graph is the reference

**Fig. 7:**
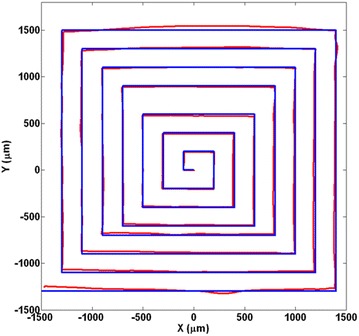
Signal after rectifying (red) versus the reference (blue)

To evaluate the mapping performance after the coordinate rotation, correcting *X*–*Y* mismatches and rectifying the distortion, the laser diode mounted on the 3D precision moving stage was used to generate various laser traces onto the PSD. In Fig. 6, a meander pattern was used to scan the whole surface of the PSD using the micro-manipulator as the reference. It became clear that the distortion is very noticeable. Using the above-mentioned corrections on the previous $X_s$ and $Y_s$, the rectifying can be applied. The result as the rectified graph that is presented in Fig. 7 shows a very good mapping with the reference. It was measured that the mapping errors in *X* and *X* are < 20 μm which means < 2%. The results clearly verify the good mapping performance by using the developed methods.

The meander pattern presents an acceptable distortion rectifying, but it is actually a graph that just one of the parameters of *X* or *Y* changes at a time. To scan the biggest area as possible of the PSD surface, an Archimedean spiral was used. This spiral can be shown using this formula:

8 $$\begin{aligned} x^2+y^2=a^2\left( \tan ^{-1} {\frac{y}{x}}\right) ^2 \end{aligned}$$

**Fig. 8:**
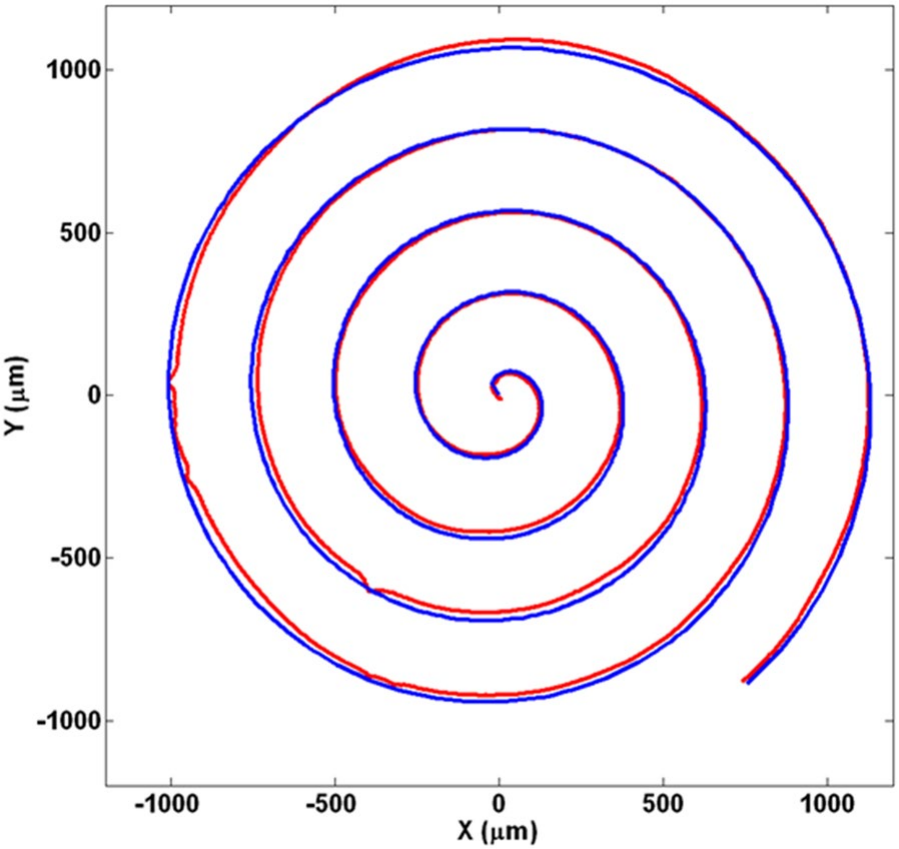
Archimedean spiral was used to verify the results. Blue is the reference and the red is the rectified signal

The result presented in Fig. 8 verifies the previous finding, and both mapping errors in *X* and *Y* within the range of 1 mm are < 2%.

### The scanning PSD microscopy

After achieving a PSD system with high reliability and accuracy, a new scanning PSD microscopy can be extended. The microscopy is based on the vanishing effect and multi-channel photocurrent feedback mechanism of the accuracy improved PSD. In this section, we will detail the findings on this new microscopy.

#### Photocurrent output and vanishing effect

**Fig. 9:**
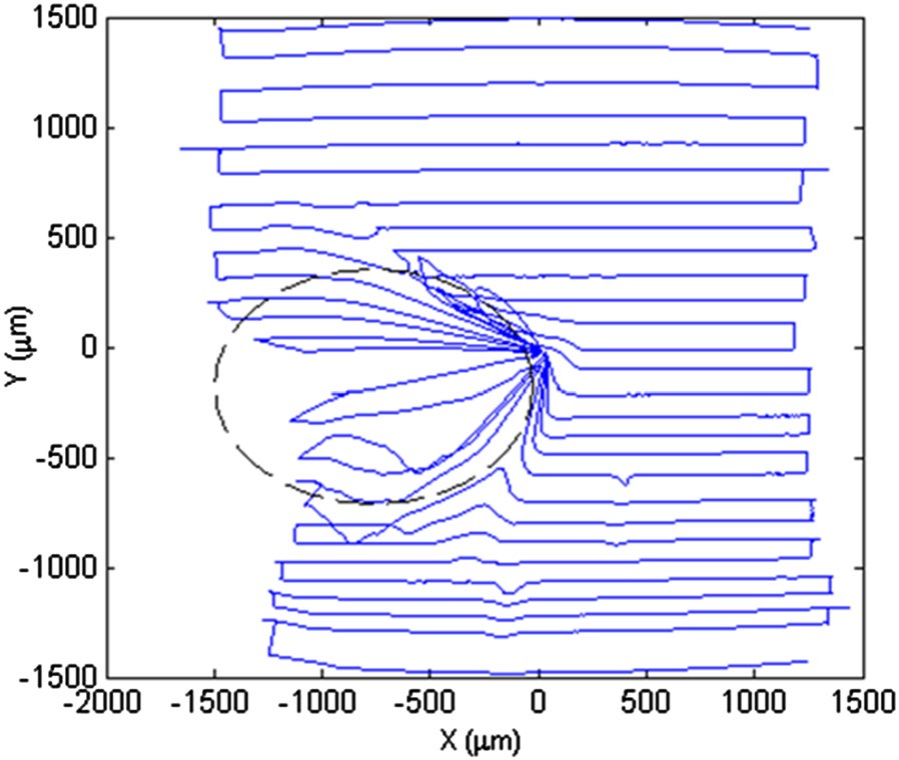
The broken lines represent a small object that was placed on the PSD. The effect can be seen as blue lines

After different tests, it became clear that the effect of blocking the laser beam can be determined by processing each of the 4 channels of the PSD individually. It was found that when there was a laser beam on the PSD, regardless of the position of the beam, each channel gives an output voltage of less than zero (because of the inverters in the PSD board), but when the light was blocked, they all quickly jump to zero. This effect is easily shown in Fig. 9.

Based on this finding and the measurement accuracy improvement methods, a scanning PSD microscopy is proposed and preliminarily implemented. The mechanism of the microscopy is based on processing 4 photocurrents from 4 input channels A, B, C, and D of the PSD and the developed accurate PSD mapping methodologies. Different methods were used to determine the exact point that the blocking of the laser occurs.

#### Three methods for finding blocking positions

**Fig. 10:**
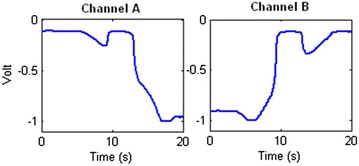
The inverse correlation and jumping to zero can be seen here. The laser has started passing an object from second 10

*Method 1* The first and the most accurate method is to monitor the smallest changes in two channels that are on opposite sides of the PSD. Using the property that was discussed in “The lateral effect PSD” section, it is known that these channels have inverse correlation meaning that when one of them increases, the other one decreases. On the other hand, from the previous section, it is known that when an object blocks the laser beam, each channel immediately translates to zero. This means they no longer have an inverse correlation. These facts are presented in Fig. 10 and are used in the first method.

The program works as it starts monitoring each channel from the beginning and as it determines that the channels are no longer in an inverse correlation, and it marks that point as the starting point of the block and, with a similar approach, finds the stopping point. By calculating these two points, it can correct the data in between and gives us the size of the object. This determination can be found out easily with this formula:

9 $$\begin{aligned} ((A(i)-A(i-1)) \times ((B(i)-B(i-1)) < 0 \end{aligned}$$

Notice it has been supposed that each current is uniform and changes smoothly, but in practice, it is not the case. As a result of this, the smallest noise can ruin this method and give a false positive. To overcome this problem, the program first finds a peak in one of the channels and then goes back to find the starting point. Unfortunately, this approach makes the first method unusable in online plotting as it needs to go back and forth in time. That is the reason for implementing the second method.

**Fig. 11:**
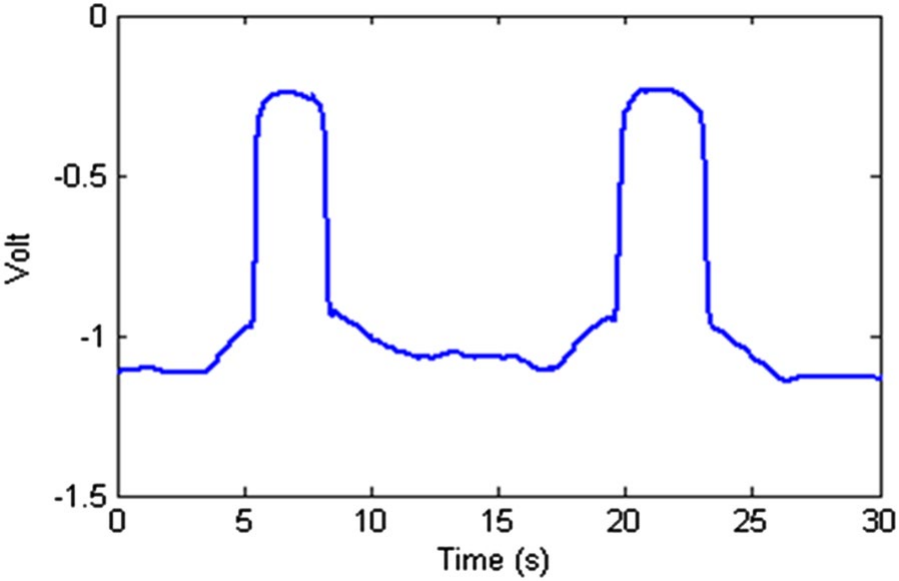
Two passes leave a clear effect on A+B

*Method 2* The second method determines the starting and stopping points by monitoring *A* + *B*. The reason is, as it is shown in Fig. 10, the inverse correlation between two channels can eliminate each other when added together in normal situations, but when a block occurs, the addition of these channels can add up resulting in a bigger peak. This method is a bit less accurate as an exact specific point cannot be determined for every case, but the advantage is that it can be used to correct the data online and generate the plots simultaneously. In this method, each time that *A* + *B* becomes bigger than a specific set number (in this case, − 0.9), a pass is marked and the starting and stopping points of the block can be extracted easily. Figure 11 presents the effect of two passes on *A* + *B*.

The second method covers most of the situations unless the object is transparent. In such a case, the laser beam would not be blocked completely and the result would be just some disturbance. Monitoring *A* + *B* does not help as it does not clearly become bigger than a set point, so the third method was designed.

**Fig. 12:**
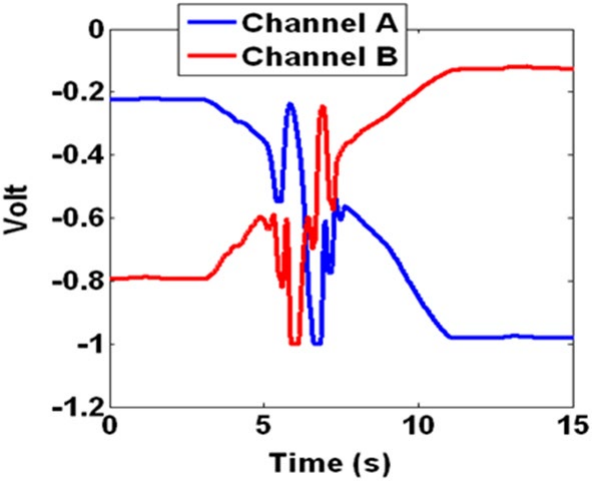
A pass of the laser over a transparent glass shows only a disturbance

**Fig. 13:**
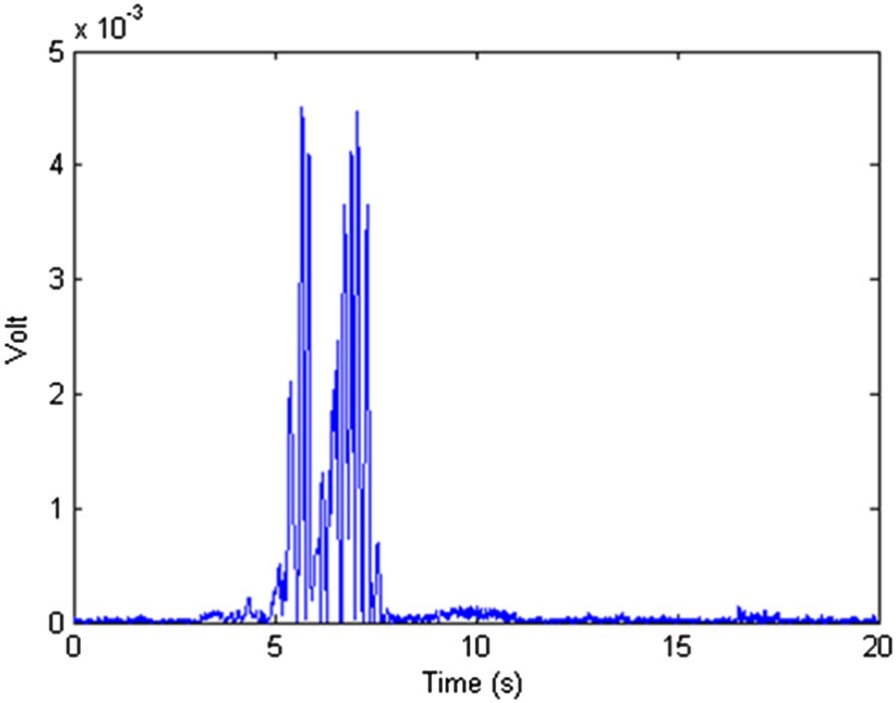
The derivative of *v* shows a disturbance from second 5 meaning a pass that lasts for about 2 s

*Method 3* The third method uses the fact that when an object, even a transparent one, blocks the laser beam, there would be a disturbance in both *A* and *B*. To monitor this disturbance, the derivative of *A* + *B* was used to find the point that *A* + *B* starts changing rapidly. These sudden changes in *A* + *B* determine a disturbance and consequently a pass. Figure 12 shows both channels *A* and *B* when the laser passes a transparent object. Figure 13 shows that the derivative of *A* + *B* has some considerably big peaks at the same time. This is because of the fact that it is showing the sudden changes in *A* + *B* which is a result of the disturbance in each of the channels.

In the end, all three methods were used to find the starting and stopping points of each block. Based on these times and as the *X* and *Y* positions of the beam for any given time is known, the program can calculate the distance that the laser beam was blocked meaning the dimensions of the object.

#### Measurements using the scanned results

**Fig. 14:**
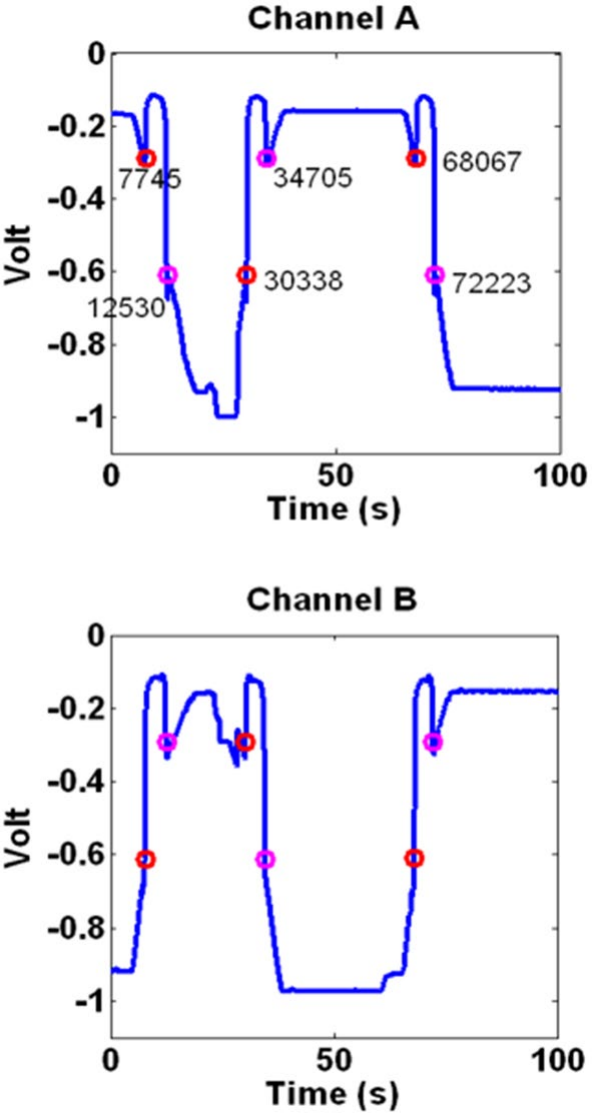
The program finds the starting and stopping points for each passes of the laser beam over the object. Here there are three passes over the object

At this point, we have all the requirements to measure the dimensions of an object. An experiment was designed to verify this ability. In this experiment, an SMD capacitor (1.57 mm × 3.05 mm × 1.57 mm) was placed on the PSD surface. The laser performs a basic scanning and passes the object three times and the program uses the above-mentioned methods to find each blocking of the laser beam. The results and measurements for both channels A and B are shown in Fig. 14.

**Fig. 15:**
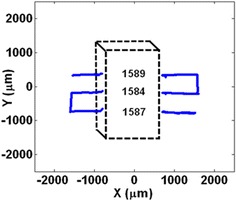
The program outputs an *X*–*Y* plot of the path that laser beam has travelled. Broken lines were added later to represent the object that was on the PSD surface

Using the time of the starting point and stopping points of each pass, the program also shows the passes on an *X*–*Y* plot alongside the measurements of the object dimensions. This is shown in Fig. 15. Note that in this figure, broken lines were added later to represent the actual position of the object on the surface of the PSD.

After extensive experiments with different objects, it was determined that the measurements are promisingly accurate. Repeating the result gives a standard deviation of < 6 μm, and the error in the full range is < 4%. It should be noted that the diameter of the laser beam we used is about 1 mm itself. So by using a laser that is capable of producing a smaller beam and focusing it properly, the measurements could improve significantly.

**Fig. 16:**
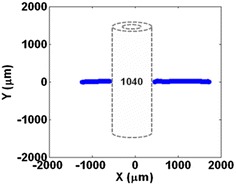
*X*–*Y* plot of the laser beam when passes over a transparent glass micropipette. Broken lines were added later to represent the object that was on the PSD surface

As it was discussed before in “The scanning PSD microscopy” section, there is a method for performing the same measurements for transparent objects. To verify that, another experiment was designed. In this experiment, a glass micropipette with an outer diameter of exactly 1 mm was placed on the surface of the PSD and the laser beam scans the surface once from side to side. By monitoring the derivative of *A* + *B*, the pass can be identified and is shown in Fig. 16.

Extensive experiments were conducted to measure the size of the transparent glass micropipette  and it proves that the measurements are repeatable with a small error as reported in prior experiments, with a standard deviation of < 6 μm. This shows that even transparent objects can be measured with this method. It is very important to note that these experiments are just preliminary proof-of-concept examples to test the abilities of the system and introduce a new method of microscopy. There are some problems with these experiments that make the final conclusion on the accuracy a little dubious. For example, these experiments were done without a good certainty that the objects are perfectly parallel to the channels. A slightly rotated object can cause the system to find different values for its dimensions as the laser beam is now not perpendicular to the sides of the object and it is more like a diagonal path that enters one side and exits another one with an angle. Another problem is that in these experiments, we used SMD resistors as an object and the dimension of different resistors might not exactly follow the suggested dimensions of the datasheet. Others factors might influence the result too. But the fact that the results are repeatable with very small error and standard deviation proves that the system is promisingly working.

## Adaptive local scanning

In this part, we address these open problems and develop and implement a comprehensive and adaptive local scanning algorithm that is capable of rapidly and accurately scanning the indiscrete, one-piece connected objects with various forms and shapes such as open or closed curves, loops, intersections or bifurcations. More importantly, our algorithm and method do not require any prior information about the scanned objects. In addition, compared to other existing local scanning methods that need a preparation or an initial scanning to know some prior information of objects before a regular local scanning, our method does not rely on any prior information about objects but points scanned online during the scanning. This will help to improve the scanning efficiency significantly. In other words, our developed method is intelligent and is more similar to a circumstance of a micro-mouse intelligently searching and finding the path. That is, the controlled micro-mouse solves a maze by keeping tracking where it is, discovering walls as it explores and mapping out the maze instead of having an already prepared map of the maze and just going through it.

In our algorithm and method, the first stage is to initially search the object in the scanning area. The initial search method is to help find one single point on the object. The initial search may be restarted in new areas if no point could be reached in the original area. Once the initial search path meets any point of the object, the second stage will start our adaptive local scanning from the scanned point. At the beginning, a sinusoidal scanning pattern with the set highest frequency and amplitude is applied toward an immediately calculated direction. As more new points of the object are being discovered along the direction, the scanning frequency and amplitude will be adaptively adjusted based on the object’s curvature and thickness that can be predicted using previously scanned points. When abnormal points represent intersections and bifurcations on the object, a new local scanning will be started from those points. Once the local scanning is finished (no new point is found in the region of interest), the final stage is to use a parametric Fourier curve fitting method to optimize the scanned points. The fitting method helps to improve scanning accuracy by minimizing the scanning error to almost half of the original error. By implementing these stages combined with other developed algorithms such as “preventing double scanning,” “determining the side of a curve” and “returning from a lost tracking,” the results validate the high performance of the developed comprehensive and adaptive local scanning method. The algorithms and method are being extended to a new scanning PSD microscopy system proposed by us [20–22]. Other applications include the fast AFM, SPM or laser scanning.

### Initial search scanning pattern

**Fig. 17:**
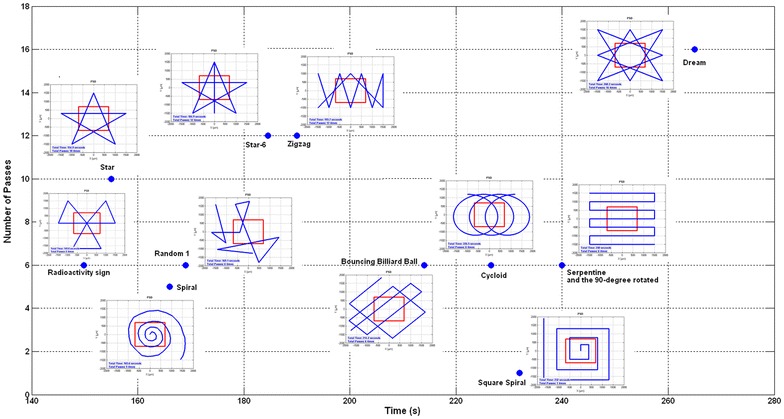
The most famous scanning patterns were tested by a simple rectangle object to find differences in scanning time and times of intersubsection

The first stage of our scanning needs a very fast and effective scanning pattern to find the unknown object. It is required to search as many areas as possible in a very short time. As just one contact with the object is needed for this stage, the pattern should be widespread to increase chances of intersecting the object. There are many patterns used for different applications [23–26]. To find the best pattern, a simulation program was firstly developed and plenty of different patterns were tested. The program has various capabilities. Any pattern or object can be defined easily in the program. The program then simulates the pattern and measures how long it would take the micro-manipulator to draw the pattern and finally how many times the pattern has passed the object edges. This gives us a good scale to find out which pattern is faster and which pattern can scan more. A representation of this simulation is shown in Fig. 17. Note that, besides a rectangle object, various objects have also been tested to prove the best pattern (i.e., Star-6).

An efficiency factor is defined as the number of passes over the whole time that a pattern takes. Also numerous patterns and different objects were examined. Finally, based on the efficiency factor, it was determined that Star-6 (a pentagram with one extra line) is the most efficient pattern. Table 1 shows the efficiency factor for different patterns in Fig. 17. To get the best pentagram, a pentagon inscribed a circle with radius *R* (that is half of a side of the scanning area) is needed.

**Table 1:** Comparison between different patterns in Fig. 17

Pattern	Time	Passes	Efficiency factor
Star-6	185	12	6.49
Star	155	10	6.46
Zigzag	194	12	6.2
Dream	268	16	5.96
Radioactivity	150	6	4.0
Random 2	219	8	3.56
Random 1	169	6	3.55
Spiral	166	5	3.02
Bouncing billiard ball	214	6	2.8
Rotated serpentine	240	6	2.5
Serpentine	240	6	2.5
Square spiral	232	1	0.43

Each side of the pentagon that inscribed a circle with radius *R* is defined with Eq. 10:

10 $$\begin{aligned} 2 \times R \times \sin \left( {\frac{\pi }{N}}\right) \end{aligned}$$

where *N* = 5 is the number of sides.

A simple pentagram pattern would find any object that has the same shape as the scanning area and is bigger than 12% of the whole area. But to be sure that smaller objects would not be missed, a second rotated Star-6 pattern will be performed after the first one. This would continue with a third scaled down one to be sure that even the smallest object would not be missed. By using all these sequences, an object should be smaller than 4.2% of the scanning area to be missed. Notice that as soon as one of the patterns has an intersection with the object, the algorithm would not continue the other ones to save time as no other scanned point is necessary.

### Adaptive local scanning methods

#### Defining a sinusoidal scanning pattern

The outcome of the previous stage is to reach the first point of the object. Once the first point is met, a sinusoidal pattern scanning will be started from this point. To map a sinusoidal pattern, a line is needed and to get a line a second point of the object should be found. To achieve this, a circular search with the first point as the center would be performed to find the second point. This circle would eventually intersect the object, giving the second point of intersection. The circle is defined in Eq. 11:

11 $$\begin{aligned} 0\le \,& {\text{ang}} \le 2\pi \nonumber \\ {\text{Circle}}\,X(ang)= \,& {\text{Intersect\,Point}} + {\text{Radius}} \times {\text{cos(ang)}} \nonumber \\ {\text{Circle}}\,Y(ang)= \,& {\text{Intersect\,Point}} + {\text{Radius}} \times {\text{sin(ang)}} \end{aligned}$$

By having these two points and using extrapolation, the first tangent vector of the object can be found. This part has been implemented in MATLAB^®^, but the ready-to-use interpolation functions of MATLAB can be sometimes bothersome. Instead, adding unit vector to one of the points to get the line was used and it is defined as follows:

12 $$\begin{aligned} \hat{u}= \,& {\frac{u}{|u|}}\nonumber \\ {\text{Line}}\,X(k)= \,& k \times \hat{u}_x \nonumber \\ {\text{Line}}\,Y(k)= \,& k \times \hat{u}_y \nonumber \\ \forall k= \,& 1,2,\ldots ,n \end{aligned}$$

Using this tangent vector, a sine wave is mapped to the line to get the scanning pattern. The sine wave is given as:

13 $$\begin{aligned} x(t)=\hat{x_{\text{d}}}(s(t))+A \sin (\omega s) q(s(t)) \end{aligned}$$

where the amplitude *A* and frequency $\omega$ are predefined values for the first scanning pattern. They may adaptively change to follow the predicted form and shape of the object. The details will be described in later sections. Each time that the pattern intersects the object, a new point has been found, and by using this new point, a new tangent vector and scanning pattern would be defined. This would let the pattern to follow the object very smoothly as it turns. The algorithm also monitors the time that pattern takes between two consequent points. Based on this, the amplitude would increase or decrease to match the object and save more time by not using a too high amplitude when it is not needed.

**Fig. 18:**
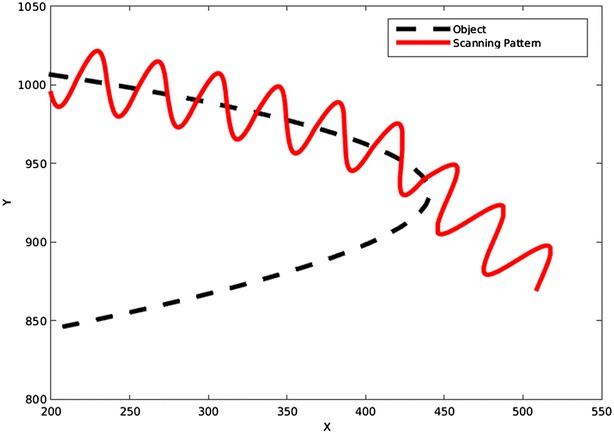
This figure shows that a constant frequency can miss the object even if the path is corrected with every new point

**Fig. 19:**
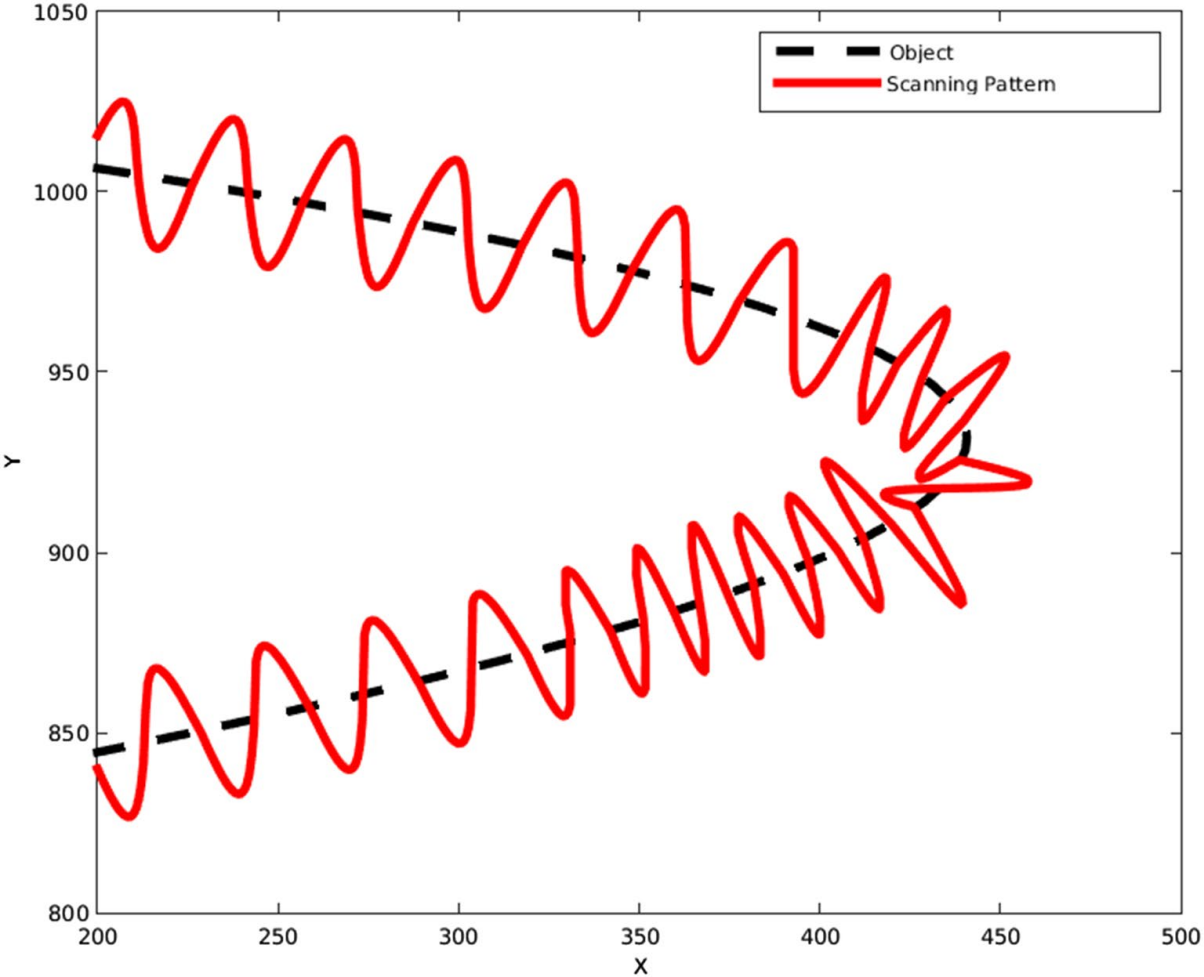
Here it illustrates that by monitoring changes in the angle of tangent vector and updating the frequency accordingly, this problem would be solved

The problem may arise that if the frequency does not change, it is likely that the pattern misses the object around a very sharp turn. This problem is shown in Fig. 18. To solve this, the frequency needs to increase when the pattern is approaching a sharp turn. To determine a sharp turn beforehand, the algorithm monitors the changes in the angle of the tangent vector for each point and compares it to the same angle of the previous points. This would let the algorithm to predict a sharp turn based on previous information. For example, the angles of the tangent vector for the few last points of Fig. 18 before the curve are: 348.4°, 343.3°, 344.5°, 338.5°, 326.8° and 309.3°, so the differences between each two consequent points are: 5.1, 1.2, 6.0, 11.7, and 17.5. By just looking at these numbers, it is obvious that a sudden change in angle is occurring, as a sequence of numbers around 4 are suddenly followed by 11.7 and 17.5. This immediate change in angle has a meaning of a sharp turn. By applying this method, the object in Fig. 18 was scanned by the new pattern. This is shown in Fig. 19.

#### Resuming the scanning for the missed points

As it was mentioned previously when the initial scanning pattern finds an intersection with the object, it would start a circle scanning and the sinusoidal pattern would start from this newly founded point. This means that by finishing scanning of this part of the object, the other side of the object is still not scanned. At this point, the algorithm would return to the starting point and resume the scanning toward the other side. Everything about the amplitude and frequency would apply to this scanning too. This way, both sides of the object have been scanned although the scanning was started from a random point—usually in the middle of the object.

One problem is that even though the frequency is changing according to the curvature of the object, there are some rare situations that the turn is so sharp and sudden that even a scanning pattern with high frequency would miss the object. In these cases, a strategy for finding the object again is needed. To achieve this, when the pattern does not find any intersection points for a given amount of time, it would start a searching scan to get back to the object. An Archimedean spiral was selected as the searching scan. This gives the benefit of scanning an area around that point in one pattern only. The Archimedean spiral is defined by the following equation:

14 $$\begin{aligned} x^2 + y^2 = a^2 \left( \tan ^{-1} {\frac{y}{x}}\right) ^2 \end{aligned}$$

or in parametric format defined as:

15 $$\begin{aligned} x{\text{(ang)}}=\, & b \times {\text{ang}} \times {\text{cos(ang)}} \nonumber \\ y{\text{(ang)}}=\, & b \times {\text{ang}} \times {\text{sin(ang)}} \nonumber \\ 0\le\, & {\text{ang}} \le a \end{aligned}$$

**Fig. 20:**
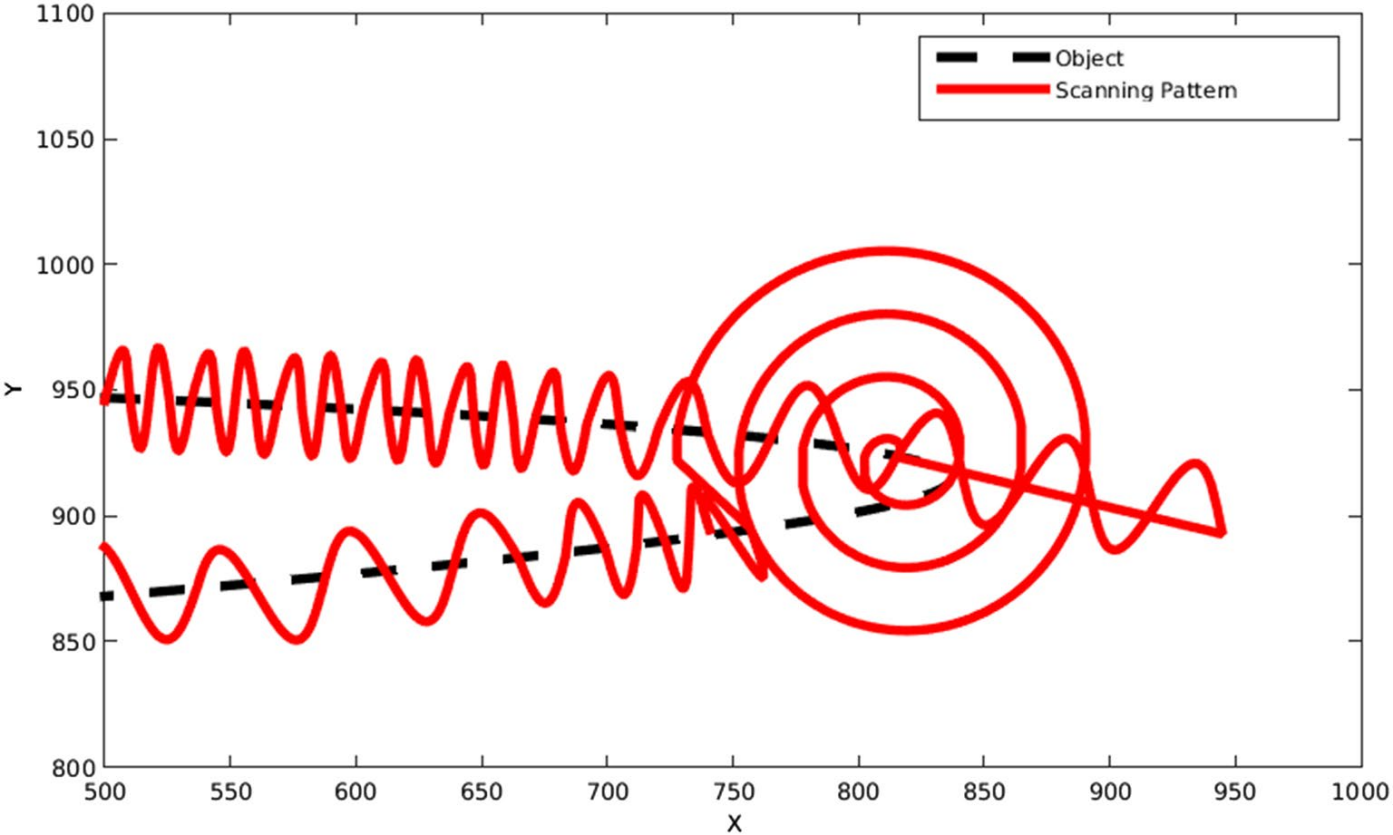
An Archimedean spiral searches for the object after the pattern misses the object due to a very sharp turn

When the searching pattern scans around a point, generally two points would be founded. One of them is the new point that the scanning would continue toward it and the other point is on that part of the object that was just scanned. By comparing these two points with all previous intersections, this can be distinguished. An example of a searching scan after a miss is shown in Fig. 20.

#### Intersections and bifurcations

Not all objects are string-like form without any intersections and bifurcations. There are examples that the object has some intersections or bifurcations such as Bifid *Y*-shaped cell that occurs most often in Gram-positive [27]. In these cases, the scanning pattern would continue toward one of the paths and the other path would remain without scanning.

Fortunately, there is a way to find these special points and return to them for another scan. Every time that the pattern reaches an intersection or bifurcation there would be a sudden change in the path that has no traces in previous points. By monitoring these sudden changes that were not expected beforehand, the algorithm can find these special points. After finishing the scanning for both sides of the object, the algorithm would return to these points, performs a searching scan and continues the scanning from these points to find any path that has not been scanned before.

**Fig. 21:**
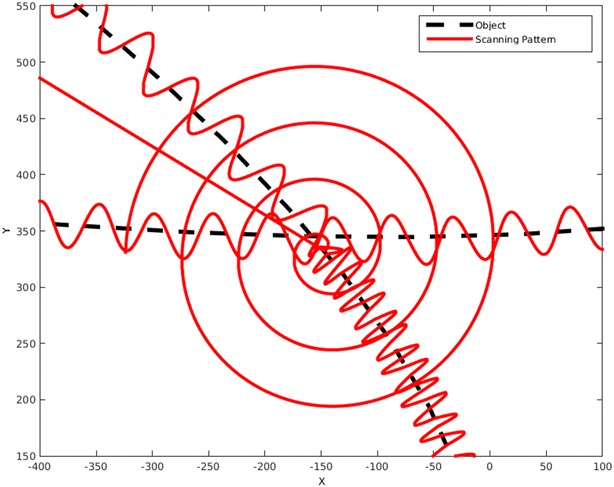
A searching scan is performed at an intersection and each path is scanned accordingly

A common occurrence in these situations is that the searching scan would find a path that has been scanned before. To prevent this, any time that the pattern intersects the object, it compares that point with all previously scanned points to be sure that this is actually a new point and this path has not been scanned before. This “New point check” algorithm would stop the program from being in a loop even if the object is just a circle. An example of the bifurcation detection and searching scan is shown in Fig. 21.

#### Scanning thick objects

When the object is very thick, the side edges are very far from each other and the algorithm would face another problem. In these cases, the tangent vector of the two sequent points is not in line with the object and is almost perpendicular to the object. This would cause a lot of problems. To solve this, when the object is thick, one side of the object is selected randomly and any further correction to the sine wave would only apply if the pattern is on the same side. By using the following method, it can be found out which side of the object is a dot located. To determine which side of the line from $A=(x_1,y_1)$ to $B=(x_2,y_2)$ a point $P=(x,y)$ falls on, *d* needs to be computed by the following equation:

16 $$\begin{aligned} d=(x - x_1)(y_2 - y_1) - (y - y_1)(x_2 - x_1) \end{aligned}$$

where if $d < 0$ then, the point lies on one side of the line, and if $d > 0$ then it lies on the other side. If *d* = 0 then the point lies exactly on the line. This is the simpler form of doing the actual method that if the point is given by the 2*D* vector $\overrightarrow{P}$ and the line end points by $\overrightarrow{A}$ and $\overrightarrow{B}$, then cross product is calculated as follows:

17 $$\begin{aligned} \overrightarrow{AB} \times \overrightarrow{AP}= \,& x_{AB} \times y_{AP} - x_{AP} \times y_{AB} \nonumber \\ x_{AB}=\, & x_B - x_A,\nonumber \\ x_{AP}=\, & x_P - x_A, \ldots \end{aligned}$$

**Fig. 22:**
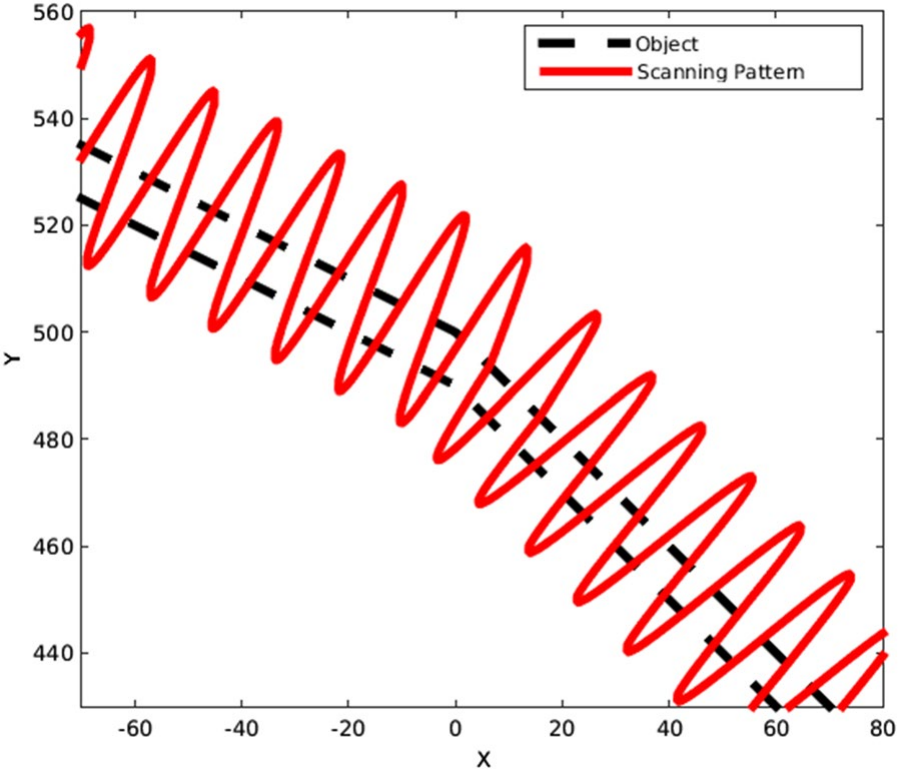
A thick object is scanned by choosing one edge as the correction side. In this figure, the upper edge has been selected automatically by the algorithm

The sign of the above will determine what side of the line the dot is located. An example of a thick object and its scanning pattern is shown in Fig. 22.

#### Flowchart of the developed algorithms and methods

**Fig. 23:**
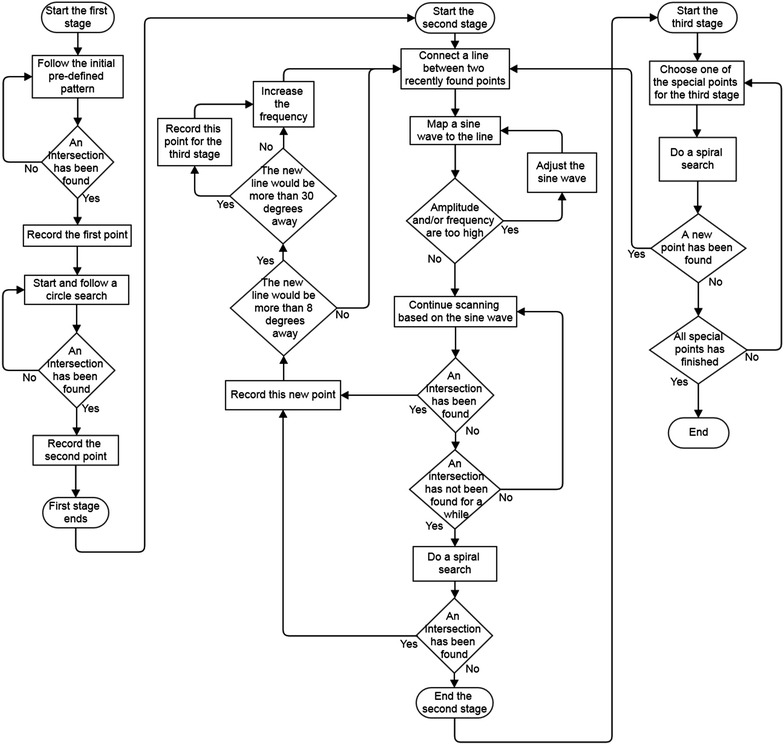
The flowchart of the algorithms and methods

The flowchart of the developed algorithms and methods is shown in Fig. 23 which is presented in three different stages. The first stage starts with the initial searching scan and continues with finding the first point of the object. Then a circle search is performed to find the second point of the object. The first stage finishes here when it has two points of the object.

The second stage begins with defining a line that passes the two recently founded points in the stage one. This continues by the mapping of a sine wave to the line and adjusting its frequency and amplitude based on the distances of the scanned points. The scanning would be resumed until a new point of the object is found. At this point, the program checks for the tangent vector of the new line which is based on the founded point. If the program determines a sharp curve, it would correct the scanning pattern based on the curvature, and if the curvature is too sharp, it would record that point for a spiral search in the third stage. The actual criteria for these decisions were found by experiment. By following the same rules, the scanning would continue until it cannot find any new points for a given amount of time which at that moment the program would perform a spiral search to be sure that this side of the object is finished and would continue to the third stage.

The third stage is when the algorithm needs to scan intersections and bifurcations again for any missed point. If by doing that, a new point is found, it would rerun the second stage based on this new point. This is to be sure that if a new path has been found at an intersection, the program would scan it thoroughly. It should be noted that this is a simplified version of the algorithm and putting all the different parts of the program in a single flowchart was not possible. When the algorithm scans all the special points and does not found any new points, the program finishes and presents the data.

#### Double scanning prevention

At this point, the algorithm can scan any object with intersections and bifurcations, but there are instances where a path that starts from an intersection and goes back to the object at a different location. The program follows every path to be sure that it has scanned all the object. But following these paths might cause the program to scan a loop over and over.

A mechanism should be implemented to prevent the program of scanning a single location twice. To do this, a function checks every newly scanned point against all the previously scanned points. If a point could be found that is within a certain distance of the new point, this can be seen as a red flag of double scanning. To be sure that it is actually scanned before—and not just a point near another part of the object—a few of the recently scanned points are examined too. In case there was no doubt that this path was scanned before, the pattern would stop and goes back to another stage of the program.

### Implementation and verification

The points that the scanning pattern finds from the object are on the outer edges of the object. To get the actual object which is between these points, an interpolation would not be suitable for the exact same reason. Instead, a parametric Fourier curve fitting with 6 terms was used as shown in the following equation:

18 $$\begin{aligned} y=a_0 + \sum _{i=1}^{6} a_i \cos (n \omega x) + b_i \sin (n \omega x) \end{aligned}$$

where these coefficients $a_0$, $a_i$ and $b_i$ in the equation are determined by the fitting function. Any number of terms more than this would result in over-fitting.

**Fig. 24:**
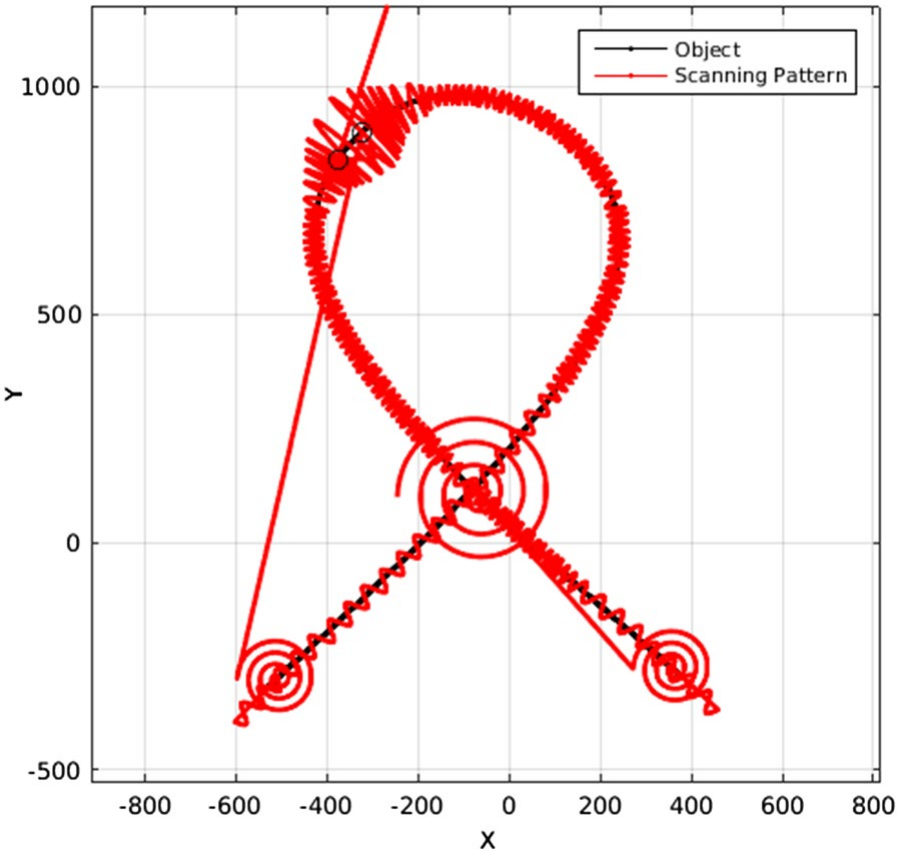
This figure shows an object and the scanning pattern

**Fig. 25:**
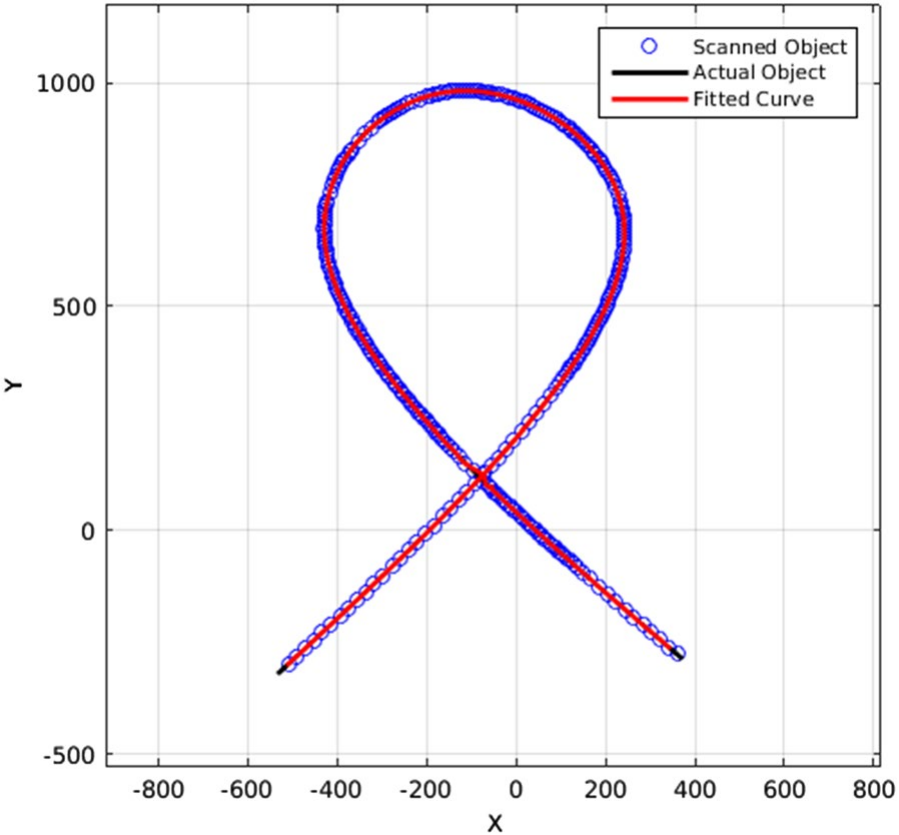
This figure displays the points that were obtained by the scanning and a fitted curve to those points. Note that as the fitted curve (in red) is very close to the actual object (in black), these two curves overlap in this figure

In addition, as the whole scanned points are obtained by a lot of sudden jumps to different positions—such as scanning each side of the object or resuming the scanning for intersections and bifurcations—it is not possible and reliable for all the scanned points to be fitted at once. To solve this, the scanned points are divided into small parts by considering the jumps and sudden changes in directions. Each part is fitted separately and the final result is obtained by simply putting these pieces together. An example of the fitting and scanning pattern is given in Figs. 24 and 25.

**Fig. 26:**
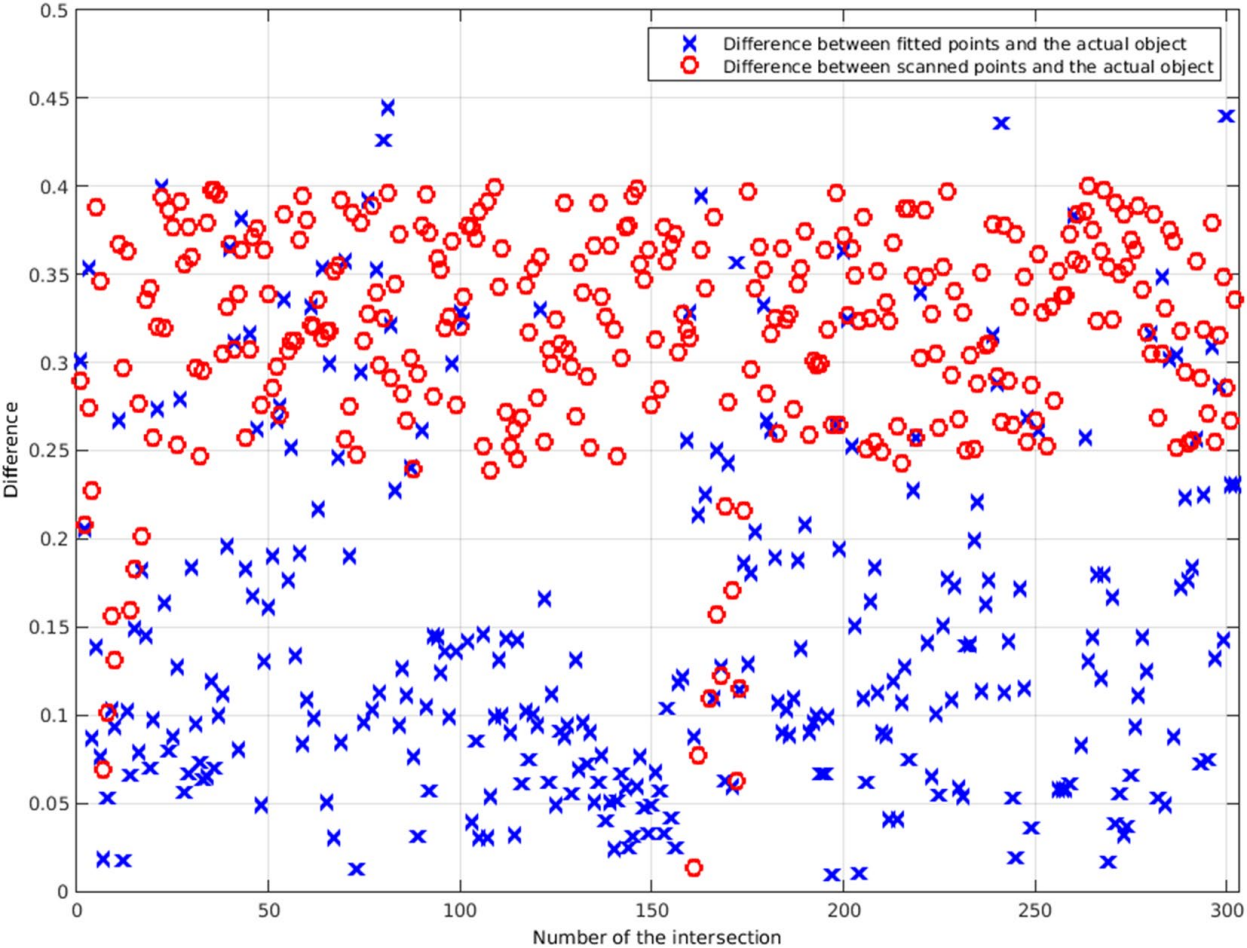
This figure shows the difference between the scanned points and the actual points of the object in Fig. 24. It also shows how the fitting has decreased the error to an almost half value

To find out what is the difference between the actual object and the fitted curve and how the fitting is helping to get a better scan, a simulation was used. Although the algorithm does not have access to the position of the actual object, it can be used after scanning to compare the results with the actual values. This comparison is shown in Fig. 26.

Figure 26 shows that the mean of the differences between scanned points and the actual object is 0.316, while the mean of the differences between fitted points and the actual object is 0.169. This shows that the accuracy has been doubled obviously. This fitting has a coefficient of determination of and only 9 points couldn’t be fitted. Also, the object is defined in a square scanning area of 4000 points by 4000 points. The mean difference of 0.169 points between final results and the actual object shows the very high accuracy of the scanning algorithm and the fitting.

To present a comparison of the improvement in the scanning time, the pattern that scanned the object in Fig. 24 needed 27,110 points for the probe to travel, while—considering the 4000 × 4000 scanning area—a traditional raster scanning probe has to travel 16 million points to achieve the same results. This means this scanning time is 0.00169 of the traditional raster scanning time which means 590.2 times faster. Obviously, this great improvement is only achievable by using local scanning and in this case, an intelligent comprehensive method that covers all the situations of different objects.

## Conclusions and future works

### Conclusion

In this study, we proposed a new microscopy system based on the PSD sensor and also developed and implemented the necessary methods and algorithm to do the scanning in time efficient manner and with good accuracy.

First, we improved the accuracy of the PSD sensor by minimizing the noises and distortions that were affecting the PSD. This was done by analyzing the noises and trying to eliminate them by various filters. We also used a rectifying method to mitigate the PSD distortion. Correction was made for *X*–*Y* mismatches and rotations and other affecting parameters. At the end, a microscopy system was proposed based on the improved PSD. Various experiments were performed to validate the accuracy of the system. A number of methods were also developed to scan solid or transparent objects. It became obvious that the system is capable of measuring the dimension of a small object with a very high accuracy and an error smaller than 2% in the whole scanning area.

The next step was to develop and implement an effective and efficient scanning method to decrease the scanning time of the area of the PSD. An adaptive local scanning method was developed to minimizing the time needed for this scanning. This is done by first using an initial scanning which would roughly find the position of the object and then a fine scanning that uses a sinusoidal pattern to scan the boundaries of the object. The pattern would follow the curvature of the object and would even recognize bifurcations and crossings. This smart algorithm would change the amplitude and frequency of the sine wave automatically to match the circumstances of the object. Thick objects can also be scanned by using another smart method. Finally, a Fourier curve fitting is employed to get even better results from the reading we got from the scanning pattern. After doing numerous experiments, it became clear that our comprehensive and adaptive local scanning method can shorten the scanning time in order of hundreds of times in comparison with the traditional raster scanning without losing any important information about the scanned object. This is due to the intelligent algorithm that can scan any object starting from any point of it.

In the verification section of the research, the differences between the actual object and the scanned object were shown. Finally to minimize the object scanning error, a Fourier curve fitting is employed to improve the scanning accuracy. Extensive implementation results demonstrate that the fitting can reduce the scanning error to an almost half value. Two examples of this reduction in scanning error were shown in the verification section.

The final result of this study is a new microscopy system that would use the PSD sensor to scan small microscale objects relying on PSD resolution. This system can scan an object as small as a few micrometers with very high accuracy and precision. The scanning accuracy and resolution can be improved by using more accurate PSD sensors and better lenses for the laser to allow a smaller light spot. That way the scanning can go to even a couple of hundred nanometers. It is obvious that the current scanning system can also be used for macroscale object too. In that case, the only limitation in size would be the size of the PSD. By using a bigger PSD, a bigger object can be placed on it and be scanned.

### Future work

The presented study shows the first steps toward the eventual implementation of a robust system approach for a new microscopy system. Although we have developed and implemented most of the algorithm and methods that are needed for this system, there are still improvements that can be done. All the current algorithm are written in MATLAB and need to be ported to C or C++ for using in an embedded microcontroller. By doing this, the system can be a stand-alone solution and would not need a computer to operate. At the end, the proposed method can benefit from more experiments to find any problem that might be left. I hope this system would 1 day lead to a very cheap and accessible microscopy system that can benefit individuals and might be used in any laboratory or classroom.
